# Functional Outcome of Isolated Hoffa Fractures Treated with Cannulated Cancellous Screw

**DOI:** 10.5704/MOJ.1707.016

**Published:** 2017-07

**Authors:** R Singh, RB Singh, M Mahendra

**Affiliations:** Department of Orthopaedics, Sardar Vallabhbhai Patel Hospital, New Delhi, India

**Keywords:** isolated Hoffa fracture, cannulated cancellous screw

## Abstract

**Introduction:** Isolated Hoffa fracture is an infrequent injury and little research has been done on this subject. The purpose of this study was to evaluate the functional outcome and complications of surgically managed Hoffa fractures with cannulated cancellous screw.

**Materials and Methods:** Between 2011 and 2014, eight isolated Hoffa fractures in seven adult patients with mean age 39.8±11.9 years (range 25-60 years) were managed with cannulated cancellous screw of 6.5mm diameter applied in anterior to posterior direction using swashbuckler and medial parapatellar approach for lateral and medial Hoffa fractures respectively. All patients were evaluated using knee evaluation score after two years or longer. Mean follow up was 28±3.8 months (range 24-36 months).

**Results:** All fractures in the eight patients healed clinicoradiologically by the 16th week with excellent result in 87.5% cases and good in 12.5% cases. By the end of union, the range of motion (ROM) of the knee was 0° to 110° except in two patients. One patient had ROM 10°-100° and other had 15°-90°. Mean knee evaluation score was 87.5±10.4. There was no incidence of non-union, infection or avascular changes in the patients or loss of reduction till final follow up.

**Conclusion:** Open reduction and fixation with two 6.5 mm cannulated cancellous screws with early mobilization yielded good functional outcome in isolated Hoffa fractures.

## Introduction

Isolated fracture of femoral condyles in the tangential plane is a rare injury and is usually accompanied with comminuted fractures of distal femoral condylar and supracondylar areas as a consequence of high energy trauma^1–5^. The exact mechanism causing this injury is still not well understood. The combinations of forces including vertical thrust and twisting may bring about this intra-articular fracture of knee^1–5^. Also possibly, the direct impact to this area with some abduction component causes this injury^6^. The Hoffa fragment more commonly emerges from the lateral femoral condyle as it is principally involved in receiving direct or oblique impact with flexed knee^6^. This fracture is intrinsically unstable owing to bony instability and muscular pull^5^. As conservative treatment yields poor result, anatomical reduction and firm internal fixation is recommended for the management of this fracture^6^. We conducted this study to analyse the clinico-radiological and functional outcomes along with complications of surgically managed isolated Hoffa fracture with cannulated cancellous screw.

## Materials and Methods

Seven patients with eight isolated Hoffa fractures in adult knees between August 2011 and June 2014 (5 lateral and 3 medial Hoffa fragments) were included in the study. The mean age was of 39.8±11.9 years. One patient had bilateral Hoffa injury involving medial condyle on both knees. All patients were evaluated with standard AP, lateral radiographs and CT scans. Patients with associated fractures, pathological Hoffa fractures or with previous history of fracture to the ipsilateral lower limb were excluded from the study.

**Fig. 1:**
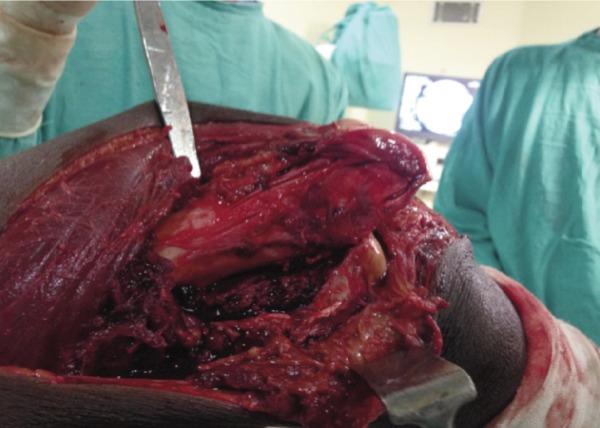
Lateral approach exposing entire distal femoral condyle and fracture fragment. Quadriceps belly was spared and reflected medially. The incision used would not impede with the future knee arthroplasty.

All coronal fractures of the lateral femoral condyle were exposed by the swashbuckler (modified anterior) approach (Fig. 1) and the medial para-patellar approach was used for medial side Hoffa fracture. Fixation was carried out using 6.5 mm cannulated cancellous screws, inserted from nonarticular surface of femur or countersinking done if inserted from the articular surface. At least two screws were used with additional screws if necessary to augment the fixation. Post-operatively long knee braces were used for immobilization and non-weight bearing range of motion (ROM) exercises were started as soon as the patient was comfortable enough. Weight bearing was allowed after six weeks. Initially partial weight bearing was allowed followed by gradual progression to unsupported walking over a period of time (12-16 weeks).

## Results

**Fig. 2:**
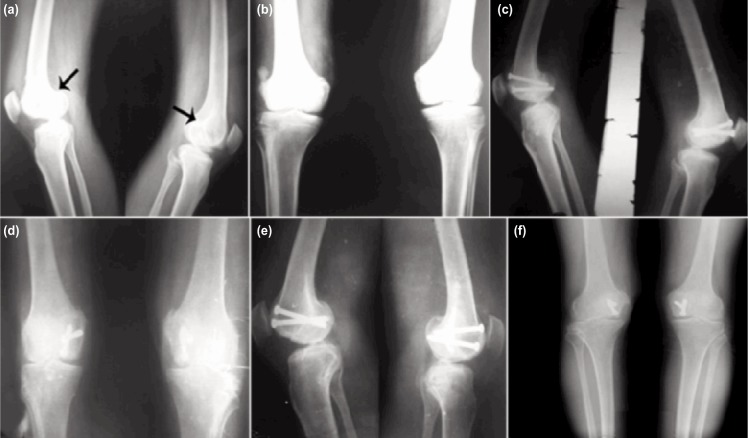
(a, b) Case with bilateral Hoffa fracture involving medial condyle on both side with (black arrow), (c, d) Plain radiographs performed at 8 weeks showing fracture union and (e, f) Radiological union was achieved by the end of 16 week.

Five male and two female patients with five lateral side and three medial side Hoffa injury were included and evaluated in our study (Table I). Mean surgical delay was 3.5±2.5 days (range 1-7 days). By 16 weeks, all fractures had united both clinically and radiologically (Fig. 2). There was no superficial or deep infection, non-union or avascular necrosis in any patients till last follow-up. There was no implant irritation requiring its removal in our study.

**Table I:** Demographic data of patients

Patient no.	Sex/Age (years)	Side	Femoral condyle	Surgical delay	Range of motion (degrees)	Knee Evaluation Score
1	F/42	Right	Lateral	1 day	0-110	90
2	M/25	Right	Lateral	7 day	0-120	95
3	M/37	Left (bilateral)	Medial	3 day	10-160	95
4	M/37	Right (bilateral)	Medial	3 day	0-100	80
5	M/45	Left	Lateral	3 day	0-110	90
6	F/38	Right	Medial	3 day	0-110	95
7	M/60	Left	Lateral	7 day	15-90	65
8	M/30	Right	Lateral	1 day	0-120	90

**Fig. 3:**
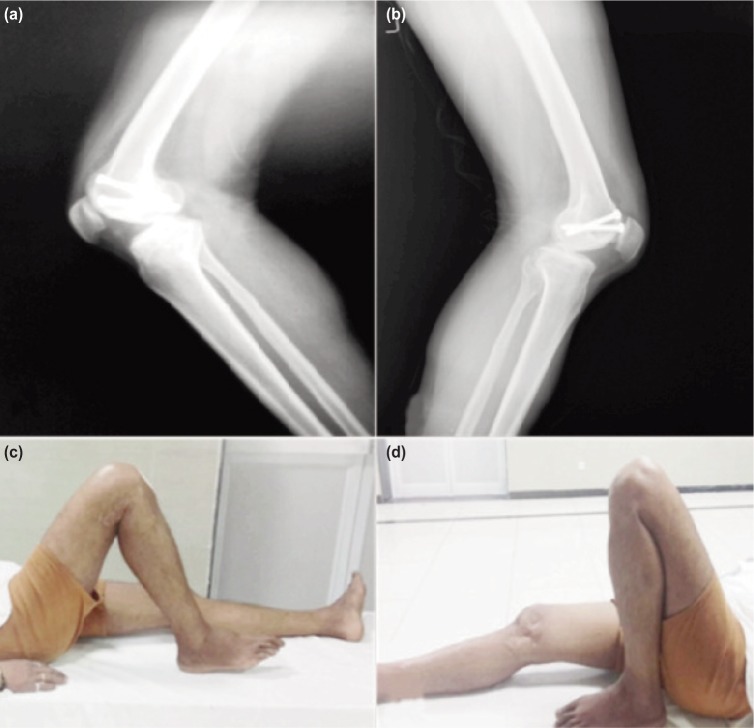
Radiographs at 36 month follow up for (a) right and (b) left knee. Functional outcome by the end of 36 month were ROM in (c) right knee was 0-125° and in (d) left was 10-160°.

All patients had at least 0°-110° range of motion except two patients (Table I). One patient had an extension lag of 10° and ROM was 10° to 100° and the other, with knee osteoarthritis and associated co-morbidities, had ROM of 15°-90°. Average follow-up of 28±3.8 months with long term follow-up of 36 month was available for three knees including the patient with bilateral Hoffa fracture (Fig. 3). Mean knee evaluation score was 87.5±10.35 (range: 95-65, Table I) while an excellent result was observed in 7 knees (87.5%) and good in 1 knees (12.5%).

## Discussion

Isolated Hoffa fracture is an infrequent injury and commonly emerges from lateral femoral condyle in comparison to medial condyle. Most authors are uncertain about the exact mechanism leading to this injury. These fractures commonly occur following motor vehicle accident^6,7^. In this study, six out of eight Hoffa fractures were seen in motor vehicle accidents with direct impact on the flexed knee. Lewis *et al* advocated that axial loading of lateral femoral condyle in a knee flexion of 90º or more with some component of abduction produces this shear fracture^7^. Four out of seven patients were riding motorcycle and had collided with motor car, with the knee in flexion in their study^6^.

The fracture was dealt with based on the principles of intraarticular fracture management^7,8^. Operative fixation warranted a good functional outcome. Non-union and malunion have been reported when Hoffa fracture was managed conservatively^6,7^. Various authors described varying exposures for these fractures. Shi *et al* favoured an interval between iliotibial band and biceps tendon and Liebergall *et al* described Gerdy’s tubercle osteotomy to improve fracture visualization and reduction^9,10^. With a more posterior approach there is a risk of neurovascular injury. We used the swashbuckler approach (modified anterior) with lateral parapatellar arthrotomy for lateral Hoffa fracture and medial para-patellar approach for medial Hoffa fracture. We ascertained that these approaches yielded ample visualization obligatory for optimum reduction and fixation with multiple cannulated cancellous screws. Lately some authors have shown promising results using arthroscopically assisted internal fixation of Hoffa fragment and advocate quicker recovery but it is still difficult to say that arthroscopically aided management had any definite benefit over orthodox open reduction^11–13^.

Hak DJ undertook the biomechanical assessment of internal fixation constructs using 3.5mm and 6.5mm screws and their data indicated that a double 6.5mm screw provided meaningfully firm construct and at least two 3.5mm screws should be used to approximate the biomechanical stability of single 6.5mm screw^14^. Jarit *et al* and Arastu *et al* show that fixation with postero-anterior oriented lag screw was biomechanically superior compared to antero-posterior oriented screw^15,16^. However, it is difficult in clinical practice as it involves more soft tissue dissection and putting the posterior neurovascular structures at risk. We used at least two 6.5mm screws in the anterior-posterior direction as it was convenient with the approach and we did not come across any implant related issue upto the last follow up.

As far as we are aware, the reports of Lewis *et al* on seven lateral Hoffa fractures and Shi *et al* on 12 isolated Hoffa fracture represent the largest single group of patients studied^6,8^. Lewis *et al* utilized two cancellous screws and Shi *et al* augmented screws using locking plates and both showed good to fair functional outcome.

Limitation of this study is the small number of cases and relatively short follow-up period. We wish to accumulate larger number cases with long term follow up for a better evaluation and detailed analysis in future.

## Conclusion

Swashbuckler and medial para-patellar approaches provide good exposure for reduction and fixation of these fractures. Open reduction and stable internal fixation of these coronal plane fractures lead to a good functional outcome. Two 6.5mm screws make the construct stable for early mobilization.

## Conflict of Interest

The authors declare no conflicts of interest in the preparation of the manuscript.
